# Are Ants Good Organisms to Teach Elementary Students about Invasive Species in Florida?

**DOI:** 10.3390/insects14020118

**Published:** 2023-01-24

**Authors:** Sara Zollota, Patricia Perez, Jenna Allen, Tori Argenti, Quentin D. Read, Marina S. Ascunce

**Affiliations:** 1 Microbiology and Cell Science Department, College of Agricultural and Life Sciences, University of Florida, Gainesville, FL 32611, USA; 2 School of Natural Resources and Environment, College of Agricultural and Life Sciences (CALS), University of Florida, Gainesville, FL 32611, USA; 3 Biology Department, College of Agricultural and Life Sciences (CALS), University of Florida, Gainesville, FL 32611, USA; 4 USDA-ARS Southeast Area, Raleigh, NC 27606, USA; 5 Emerging Pathogens Institute, Department of Plant Pathology, University of Florida, Gainesville, FL 32611, USA; 6 USDA-ARS Center for Medical, Agricultural and Veterinary Entomology, Gainesville, FL 32608, USA

**Keywords:** ants, invasive species, elementary school, awareness, active learning, native species

## Abstract

**Simple Summary:**

Ants are charismatic and easy-to-find organisms that may be good candidates for teaching elementary students about invasive species. Thus, we created an educational program named “The ImportANTs of ANTs”, which includes a set of active-learning stations and activities where students can learn about ant species diversity, native and invasive species, and invasive species’ impact on the environment, a field that is usually not touched upon in a typical classroom setting. To quantify the program’s effectiveness on the students’ perceptions of ants and invasive species, we used pre- and post-surveys and analyzed response changes after the completion of the program. Using active learning approaches, we engaged the children in learning about how ants live and interact with other species, explained the differences between invasive and native species, and demonstrated how native ants benefit the environment. The survey analysis revealed an increase in the students’ positive feelings about ants and a significant increase in their awareness of invasive species’ negative impacts on ecosystems.

**Abstract:**

This study aims to evaluate the effectiveness of our outreach program “The ImportANTs of ANTs” in communicating scientific topics to elementary school children, using ants as example organisms. In this program’s first phase, we focused on the concepts of native and invasive species and how invasive species affect ecosystems. The program included various active learning approaches, including presentations, handouts, crafts, and live colony viewings. At two schools (one in rural and one in suburban areas), 210 students from 5th grade were assessed using short, anonymous pre- and post-surveys. We analyzed the students’ responses to questions from the following categories: general feelings about ants, ant knowledge, general care for the environment, general impact knowledge, and native/invasive species knowledge. The school populations displayed distinct opinion changes and knowledge gains; however, there was a significant increase in knowledge of native and invasive species in both populations. Our study demonstrates that ants are good models to teach children about the impact of invasive species. The project aims to drive universal responsibility by forging proactive attitudes toward protecting the environment and native species early.

## 1. Introduction

**“*Every kid has a bug period … I never grew out of mine*.”**

—**E.O. Wilson**

Insects are ideal flagship organisms for classroom usage in K-12 [1]. Foremost, insects represent nearly 75 percent of the Earth’s fauna [2]; between 2.5 and 3.7 million insect species exist and play essential roles in the environment [3,4]. For example, insects are a critical food source for many organisms, and as decomposers, they maintain the biodiversity of tropical rainforests and other biomes by restoring nutrients to the soil [4]. They also display morphological features that lead to their unique aesthetic appeal (e.g., the significant variation in colors and patterns of butterfly wings) and diverse life cycles [1].

Insects interact with humans in myriad positive and negative ways [1]. For example, nearly 35 percent of the world’s food crops depend upon animal pollinators, most of which are insects such as honeybees [5]. Additionally, insect products are utilized in many industries, including the medical field and cosmetics. Some insects, such as termites, cause substantial damage to infrastructure, and others, such as mosquitoes, are vectors for human pathogens. Most invasive insects are a considerable threat to agriculture and natural resources worldwide, accounting for 120 billion dollars annually in losses in the US [6]. Florida is at high risk for invasive insects due to its subtropical climate, amount of shipping ports, and significant presence of plant-based industries [7]. However, invasive species topics are not widely taught in Florida K-12 curricula. Despite the importance of beneficial and invasive insects, many K-12 teachers still hesitate to use insects as teaching tools [8]. General attitudes towards insects can be overwhelmingly negative, especially regarding stinging insects such as wasps and ants [1]. These negative perceptions may be due to the physical, behavioral, and size differences between insects and humans [9]. Due to their sheer numbers, much is still unknown about insects, and this uncertainty can be nerve-wracking for any teacher [1].

With the support of the UF Thompson Earth Systems Institute (TESI), we created an outreach educational program to teach young students about our natural environment, the organisms that live in it, and how invasive species can have detrimental effects. The program is called “The ImportANTs of ANTs”, and its goals are: (a) to encourage children to learn about native organisms, (b) to teach children about the negative impact of invasive species, and (c) to empower students to protect nature. We focused on native and invasive ants in Florida to foster emotional connections to local natural environments. Florida is considered a host spot for invasive ants, as it harbors the largest percentage of non-native ants in North America (over 50 exotic species across 25 genera) [10,11,12]. Thus, there is a critical need for public education in Florida. There are few studies on ants as flagship organisms in K-12 classrooms, with only one study done on the little fire ant, *Wasmannia auropunctata* (Roger) in Hawaii [13]. As many studies have shown a link between public awareness and outreach programs that aim to provide literacy on biosecurity [14,15,16,17], we hope that “The ImportANTs of ANTs” program can serve as inspiration for researchers in other geographic regions. In this study, we aim to quantify the effectiveness of our outreach program “The ImportANTs of ANTs” in communicating scientific topics to elementary school children, using ants as example organisms. To accomplish our goal, we conducted our program at two schools, one in a rural setting and the other in a more urban area, where 210 elementary students participated in different active learning activities about ants. Students answered questions before and after the program, and their responses were analyzed to quantify the effectiveness of the activities. Findings from this study can encourage teachers to use ants in classrooms and as models to teach about native and invasive species concepts.

## 2. Materials and Methods

### 2.1. Participants

We conducted our outreach program “The ImportANTs of ANTs” at two elementary schools in Florida: ME in Melrose, which is in a rural area, and LE in Gainesville, which is in a more urban area. ME and LE were visited in April and October of 2019, respectively. At ME, we presented the program to 120 students from four 5th-grade classes of about 30 students each, while at LE, we presented it to 90 students from three 5th-grade classes of about 30 students each.

### 2.2. Surveys

To assess the effectiveness of our educational program, “The ImportANTs of ANTs”, we gave each student a pre-survey (Table S1) and a post-survey (Table S2) at the beginning and end of the program, respectively. Each survey was completed in about ten minutes. The surveys contained questions organized into three main categories: Ant Interest, Ecosystems and Environment, and Ant Knowledge. At the end of the post-survey, we included additional questions about the students’ opinions of the program, their intentions after the program, and their interest in learning more. These questions were not included in the analysis. All surveys were anonymous, and no personal information was recorded.

### 2.3. Data Analysis

Upon completing the program, we grouped the pre- and post-surveys by school. The responses were manually counted and recorded. For statistical analysis, the questions were organized into five categories: Ant Knowledge, General Impact Knowledge, Native/Invasive Species Knowledge, General Feelings about Ants, and General Care for the Environment (Table 1). To assess the program’s effectiveness, we compared the categories using each school’s pre- and post-survey data. Additionally, we compared the pre-survey data between the schools, as this gave us insight into the general knowledge level of each school.

We fit two generalized linear models (GLM), one for the knowledge-based questions (including the categories Ant Knowledge, General Impact Knowledge, and Native/Invasive Species Knowledge) and one for the attitude-based questions (including General feelings about ants and General care for the environment). In the knowledge model, we modeled the probability of a correct answer (considering “No” and “I don’t know” to be incorrect). In the attitude model, we modeled the probability of a positive answer (considering “No” and “I don’t know” to be negative). In each model, we included survey (before or after), category, and school (ME or LE) as categorical predictors and all two-way and three-way interactions. We examined diagnostic plots to confirm that assumptions of homogeneous variance and roughly normal distribution of residuals were met. We estimated marginal mean probabilities of correct or positive answers for each survey (before or after) averaged across categories and schools, each survey within each category averaged across schools, each survey within each school averaged across categories, and each survey within each combination of school and category. We computed pairwise contrasts (odds ratios) between surveys within and between categories and schools. We also individually computed the contrast between schools for the pre-survey and the post-survey. Odds ratios (OR) were assessed for statistical significance using the associated z-statistic with α = 0.05. When more than one pair of means was included in a comparison, the *p*-values were adjusted with the Sidak multiple comparisons adjustment. A step-by-step summary of the analysis, including the R code used, is provided in the Supplementary Material.

### 2.4. Outline of the Active Learning Activities

This paragraph provides a general outline of the program’s activities. After the pre-survey (10 min), the program started with a general presentation about ants and their life cycle (20 min), followed by an ant craft activity (20 min). The second section focused on Native/Invasive Species and the impact of invasive species on ecosystems (20 min). We finished the program by presenting live ants (15 min), followed by the post-survey (10 min). The program duration was approximately 1.5 h, depending on the volume of questions asked by the students. 

#### 2.4.1. Ants and Life Cycle Activities

**Figure 1:**
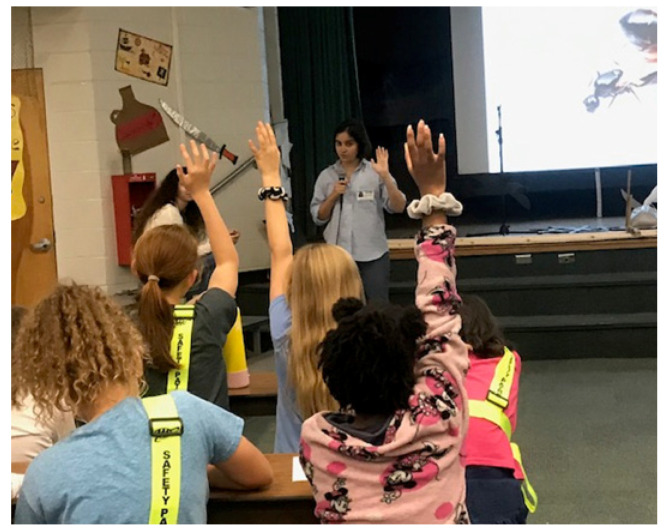
Fifth graders asking questions about ants. Picture by M.S. Ascunce.

The topics of ant morphology, ant diversity, and ant social structure were taught utilizing an electronic slide presentation that included pictures of body parts from different ant species and caste categories. Because this was our first interaction with them, we spent some time introducing ourselves and allowing the students to ask us questions. This icebreaker segment allowed us to connect with the children and create an environment for them to feel comfortable asking questions. We asked closed and open-ended questions on each slide to capture students’ attention, foster student involvement, and facilitate a positive, active learning environment. The students asked various questions, which allowed us to adapt the presentation to each class’s dynamic (Figure 1). Once the presentation ended, the students moved on to coloring sheets that illustrated the main ant body parts on an outline of a minor worker. This activity allowed the students to reflect upon and have fun with the topics from the presentation. During the coloring activity, we moved around the classroom and answered questions, which helped the quieter students to ask us questions through more personalized interaction.

#### 2.4.2. Ant Craft Activity

**Figure 2:**
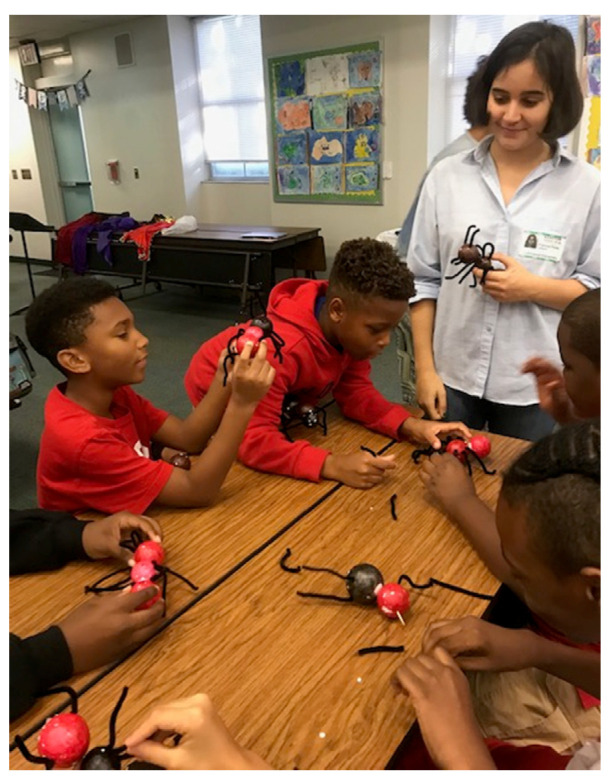
Fifth graders building ants. Picture by M.S. Ascunce.

The goal of the ant craft activity was to provide the students with a fun, hands-on way to solidify the educational concepts they learned in the previous presentation. Supplies for this craft included two (2) 2-in Styrofoam balls (one for the head and one for the abdomen), one (1) 1-in Styrofoam ball (for the thorax), toothpicks (to connect the body parts), googly eyes, and pipe cleaners (to serve as antennae, legs, and other features). For more information, see Text S1. We painted the Styrofoam balls prior to completing the activity. We taught the students how to build the backbone of the ant (head, thorax, and abdomen), but they were encouraged to customize other features, such as stingers, mandibles, and wings (Figure 2). This activity encouraged the students to take ownership of their learning and allowed them to create their unique keepsake.

#### 2.4.3. Native/Invasive Species

A short Prezi presentation introduced students to the concepts of native species and their many benefits to native environments. Initial examples of native species provided to the students were the American alligator and the red velvet ant, *Dasymutilla occidentalis* (Linnaeus), a solitary wasp. This presentation concluded with a discussion of the Florida carpenter ant, *Camponotus floridanus* (Buckley), an essential decomposer and food source in Florida habitats. We used the same questioning methods as the first activity on ants (i.e., combining closed and open-ended questions). Additionally, we asked for the students’ opinions and life experiences related to the topic, reinforcing a student-centered learning experience.

The same approach of combining a short Prezi with questions was used to introduce the concept of invasive species and their impact on ecosystems. Initial examples of invasive species provided to the students were the pythons that were accidentally released into the Florida Everglades and their negative impact on native alligators. Another example was the lionfish invading many Florida waterways and out-competing native fish. We highlighted how the Red Imported Fire Ant (RIFA), *Solenopsis invicta* (Buren), impacts Florida and much of the southeastern United States through economic, environmental, and medical costs. The presentation concluded with a discussion of how to limit the impact of invasive species, including mindful travel and pet ownership and the importance of scientific research.

While we focused on species native to Florida, we encourage other researchers and teachers interested in replicating these activities to use native and invasive species that affect their local regions. This approach builds emotional connections to local natural environments and has a long-term impact on children.

#### 2.4.4. Live Ant Farms

**Figure 3:**
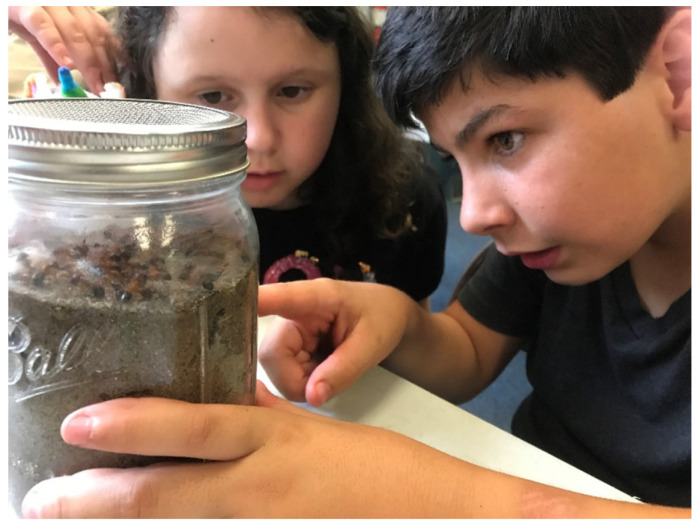
Fifth graders looking at Florida carpenter ants. Picture by M.S. Ascunce.

Observing live animals benefits students because it reinforces critical learning concepts, sparks their interest in science, and promotes pro-environmental actions [18]. For this activity, we organized the students into four groups of up to 8 children and allowed each group to observe one ant species at a time. As a safety precaution, adults handled each jar among tables. Jars were glass and had a mesh cover to prevent ants from escaping. Children could touch the jars under supervision (Figure 3). We included ant farms with the Florida pyramid ant, *Dorymyrmex bureni* (Trager), which likes open areas and sandy soils; *Trachymyrmex septentrionalis* (McCook), a native, fungus-growing ant; the Florida carpenter ant, *Camponotus floridanus* (Buckley), which can act as a house pest; and the obscure big-headed ant, *Pheidole obscurithorax* (Naves), an invasive ant species that can coexist with RIFA in disturbed Florida habitats. This independent time allowed students to reflect on the topics discussed in earlier parts of the program. Common topics of discussion included the composition of an average ant nest, differences in morphology according to caste, and ant diets.

## 3. Results

### 3.1. Pre/Post-Survey Comparisons within Each School and Averaged across Schools

**Figure 4:**
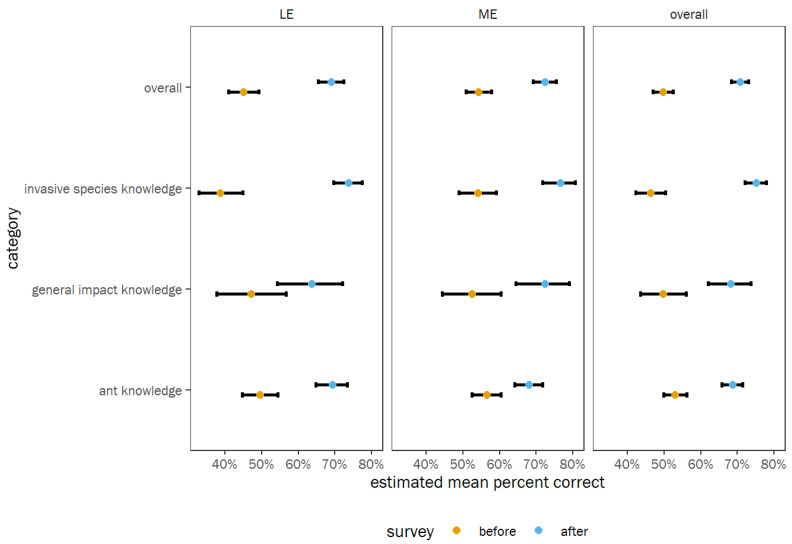
GLM’s estimates of percent correct scores for each category within the Knowledge group at each school separately, as well as averaged across categories overall and averaged across schools overall. Points show the estimated marginal means, colored by survey (before or after), and error bars show the 95% confidence intervals of the means.

Generalized Linear models (GLM) were fit to compare each category within each group: Knowledge and Attitude. A full array of comparisons is provided in the Supplementary Material, including the R code. For the Knowledge group questions, there was an increase in knowledge for all three categories at both schools (Figure 4). This is also reflected in the z-tests of the odds ratio contrasts between surveys (Table 2, Supplementary Material). In the Knowledge group analysis, there is a significantly greater proportion of positive attitude scores after the program versus before, averaged across all categories and schools (OR = 2.45 times greater odds of positive attitude scores, *p* < 0.0001). The odds ratio contrasts also indicate that knowledge about invasive species increased more than knowledge about ants (OR: 1.808, *p* < 0.001; Supplementary Material).

**Figure 5:**
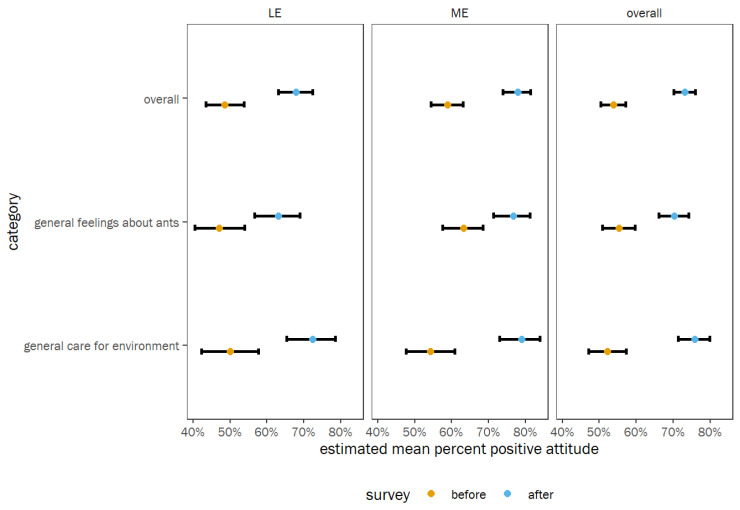
GLM’s estimates of percent correct scores for each category within the Attitude group at each school separately, as well as averaged across categories overall and averaged across schools overall. Points show the estimated marginal means, colored by survey (before or after), and error bars show the 95% confidence intervals of the means.

Regarding Attitude, there was also a strong trend in increasing positive attitude about ants and care for the environment because of the program (Figure 5, Table 2). Odds ratio contrasts indicated a significantly greater proportion of positive attitude scores after the program versus before, averaged across all categories and schools (OR = 2.35 times greater odds of positive attitude scores, *p* < 0.0001) (Table 2). When comparing the trend between question categories in the Attitude group, there was weak evidence that positive feelings about care for the environment increased more than positive feelings about ants (OR = 1/0.663 = 1.51 times greater increase in positive feelings about environment relative to ants, *p* < 0.05, Supplementary Material). However, all before versus after trends were significantly positive when looking at each category and school individually (Figure 5, Supplementary Material).

### 3.2. Pre-Survey and Post-Survey Comparisons

The odds ratio contrasts when comparing both schools during the pre-survey indicate that ME had significantly more positive attitude than LE in the pre-test for both category groups: Knowledge (OR = 1.45, *p* < 0.001) and Attitude (OR = 1.52, *p* < 0.01) (Table 2). This suggests that ME and LE students started the program with different knowledge and attitudes based on the questions provided in the survey. For the Post-surveys, ME had a significantly more positive attitude than LE only for the Attitude group (OR = 1.66, *p* < 0.01) while there were not significant differences in the Knowledge group (Table 2). This reflects that, at the Knowledge level, both schools ended up with similar gains, and that the program was able to close the knowledge gap found during the pre-survey.

General trends demonstrate that LE was able to catch up with ME in the Knowledge group with an OR of 0.85; however, the difference was not significant (*p* > 0.05) (Table 2). For the general trend in the Attitude group, ME seems to have a slightly higher gain with a OR of 1.1, which was not significant (*p* > 0.05) (Table 2). These results indicated that both schools increased in knowledge, and positive attitude, by about the same amount.

## 4. Discussion

Florida’s climate and geographic location as a transportation hub for goods and tourism have made this region a hot spot for invasive species. Raising awareness about invasive species is the first step in fighting this problem; however, invasive species are not common topics in Florida’s K-12 curriculum. To bring awareness to younger generations, we created “The ImportANTs of ANTs”, an outreach educational program which teaches young students about our natural environment, organisms that live in it, and how invasive species can have detrimental effects. In this study, we evaluated the program’s effectiveness in communicating scientific topics to elementary school children through pre- and post-surveys about the topics covered in the program. The results of this study show that we successfully improved the children’s knowledge about native and invasive species, which was our primary goal. In addition, we were able to find significant improvements in other areas, as discussed below, that allow us to encourage researchers to replicate the program in diverse geographic regions using relevant invasive species.

Because ME is located in a rural area, and LE’s setting is suburban, we wanted to determine if the student populations had a similar initial knowledge level on the topics. Comparing the pre-survey responses from each school indicated that the students at ME started the program with a higher level of knowledge and attitude, based on the questions provided in the survey (Table 2). It is possible that the ME students were more knowledgeable about these topics because invasive pests may be a more relevant discussion topic in rural areas, and children in in these areas have more chances to encounter nature. The post-survey responses indicated that ME had significantly more positive attitude than LE only for the Attitude group. Interestingly, LE was able to catch up with ME in the Knowledge group, suggesting that the program was able to close the knowledge gap found during the pre-survey. Overall data trends suggested that both groups of students experienced similar gains in knowledge levels and general feelings after the program. Comparisons of pre- and post-surveys within each school for the Knowledge and Attitude groups demonstrated that there was an increase in knowledge and positive attitudes at both schools (Figure 4 and Figure 5). These results suggest that our program fostered positive feelings about ants and their essential roles in the environment and ecosystem. Interestingly, knowledge about invasive species increased more than knowledge about ants (Supplementary Material), supporting the idea that ants are good models to teach children about the impact of invasive species. Studies conducted in regions beyond north central Florida may provide greater insight into the data presented in this study, which was collected from a limited geographic area. Additionally, other insects can serve as models in areas with other invasive species concerns. 

An important step toward insect conservation is breaking the barrier of negative perceptions that people have towards insects [19], which has partially contributed to their decline [10]. It has been posited that 40% of insect species in temperate regions may face extinction within the next 30 years due to climate change, habitat loss, and fragmentation [20]. It has also been suggested that, if ants, termites, and bees alone are eliminated from the environment, most angiosperm species will become extinct, soils will be depleted of their nutrients, erosion will significantly increase, and the tropical rainforests will die off—in other words, the Earth will eventually become uninhabitable without these vital insect species [4]. Considering insects’ immense roles in the environment, it is in our best interest to foster an appreciation for insects in younger generations. By exposing young students to the importance of insect conservation, we hope to foster a greater sense of environmental appreciation and awareness. The students’ developed stewardship could also lead to an interest in the sciences and scientific research in the future, as well as enhanced literacy on invasive threats [14,15,16,17,21]. 

Some considerations for the future of this program are its continuity and long-term effects, as well as teacher involvement. There is a lack of evidence on the long-term effects, such as sustained interest in environmental conservation or increased interest in pursuing higher education related to conservation, caused by this type of outreach program [22,23]. Programs like “The ImportANTs of Ants” only show short-term effects, partly because of their short-term duration. However, long-term implications, such as college or major choice, are challenging to assess, as they become relevant long after the program’s initial delivery [24,25,26,27]. To increase the likelihood of our program having long-term impacts, we are committed to visiting the schools every year and providing opportunities for virtual sessions on specific topics. Regarding teacher involvement, they did not actively participate in the educational activities during our initial program delivery. To address this, future classroom visits will incorporate material that directly engages teachers. Additional learning opportunities, such as teacher training sessions, may help the teachers feel more comfortable using ants and insects in their classrooms.

## 5. Conclusions

“The ImportANTs of ANTs” significantly improved students’ knowledge of native and invasive species. Although invasive species drive biodiversity loss and have drastic economic and health-related impacts, the K-12 educational system does not prioritize this topic in Florida. We hope that our work can be used to teach students about this crucial issue. Furthermore, “The ImportANTs of ANTs” can destigmatize the use of ants in classrooms and increase children’s love for ants, which may be particularly significant in schools in urban areas, such as LE.

## Figures and Tables

**Table 1:** Analysis design with questions organized into five categories distributed in two groups: Knowledge and Attitude.

**Group**	**Category 1**	**Ant Knowledge**
Knowledge	Question 1	Is there only one type of ant?
	Question 2	Do ant bodies have three parts?
	Question 3	Do ants use chemical signals to communicate with each other?
	Question 4	Do fire ants love sugar?
	Question 5	Are fire ants a problem?
	Question 6	Are fire ants an invasive species?
	Question 7	Are fire ant stings dangerous?
	Question 8	Is it safe to touch a fire ant pile?
	**Category 2**	**General Impact Knowledge**
	Question 1	Do invasive species affect natural ecosystems?
	Question 2	Does controlling invasive species cost our government money?
	**Category 3**	**Native/Invasive Species Knowledge**
	Question 1	Ants interact with the ecosystem as much as any other animal.
	Question 2	Invasive ants compete with native ants for food and resources.
	Question 3	Protecting native ants is important for the environment.
	Question 4	Do you know what an invasive species is?
	Question 5	Do you know what a native species is?
**Group**	**Category 4**	**General Feelings about Ants**
Attitude	Question 1	Do you like ants?
	Question 2	Are ants fun to learn about?
	Question 3	Are ants important?
	Question 4	Do you want to learn more about ants?
	**Category 5**	**General Care for the Environment**
	Question 1	Do you help to protect the environment every day?
	Question 2	Do you want to protect the environment and ecosystems around you?
	Question 3	Do you care about native species?

**Table 2:** Summaries of Pre/Post-Survey comparisons. Results are shown for categories in both Knowledge group (first row) and Attitude group (second row). An odds ratio of 1 indicates no change.

Comparisons	Odds	Standard	z-Ratio	*p*-Value
	Ratio	Error		
Average across group categories				
Knowledge categories	2.450	0.196	11.209	*3.7 × 10^−^* ^29^
Attitude categories	2.350	0.245	8.187	*2.67 × 10^−^* ^16^
ME/LE ratio, pre-test				
Knowledge categories	1.449	0.161	3.348	*0.000813*
Attitude categories	1.515	0.211	2.975	*0.00293*
ME/LE ratio, post-test				
Knowledge categories	1.181	0.136	1.442	0.149
Attitude categories	1.665	0.258	3.289	*0.00101*
trend comparison across all categories				
Knowledge categories	0.815	0.130	1.283	0.2
Attitude categories	1.100	0.229	0.455	0.649

## Data Availability

The data presented in this study are available in the supplementary material.

## Supplementary Materials

The following supporting information can be downloaded at: https://www.mdpi.com/article/10.3390/insects14020118/s1, Table S1: Pre-survey. Questions included in the questionary before starting the educational program; Table S2: Post-survey. Questions included in the questionary after the educational program. Text S1: Ant Craft Guideline. A step-by-step statistical analysis, including the R code used and summary of responses, are provided in the Supplementary Material.
