# Supplementary material for: Are Ants Good Organisms to Teach Elementary Students about Invasive Species in Florida?
Source: Insects. 2023 Jan 24;14(2):118. doi: 10.3390/insects14020118 (PMC9966977; doi:10.3390/insects14020118)
Supplement: Supplementary file 1 [file insects-14-00118-s001.zip › insects-2065559_R1_Text S1.pdf]

Text S1. ANT CRAFT GUIDELINE

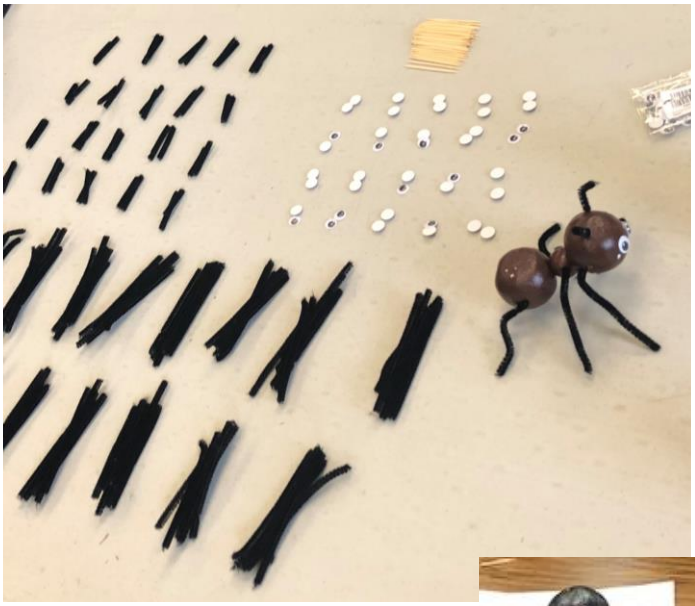

Supplies
- 2 Large Styrofoam balls
- 1 Small Styrofoam ball
- 6 large pipe cleaners
- 2 small pipe cleaners
- 2 toothpicks
- 2 googly eyes
- Optional: Extra pipe clearness for wings and mandibles
- Optional: Extra toothpick for stinger

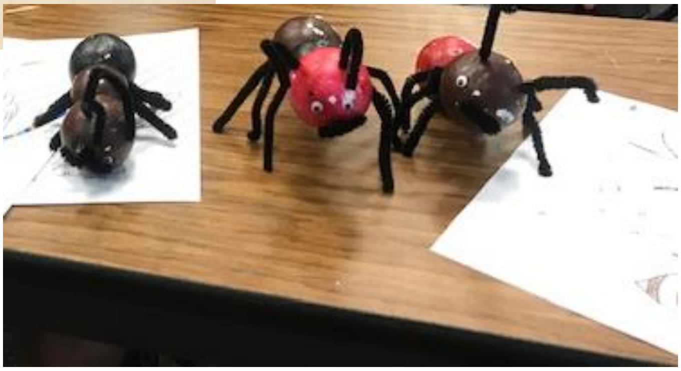

Directions

1. Use toothpicks to stick Styrofoam balls together tom make the ant body: to make a classic ant shape, put the smaller ball between the 2 larger ones.

2. Stick the pipe cleaners into the balls to make antenna and legs: remember ants have 6 legs

3. Use glue to stick the googly eyes on.

4. Feel free to be creative: Ants come in all shapes and sizes!

5. What else do ants have? Do they fly? Do they carry food? What do they use for that?
