# Supplementary material for: Are Ants Good Organisms to Teach Elementary Students about Invasive Species in Florida?
Source: Insects. 2023 Jan 24;14(2):118. doi: 10.3390/insects14020118 (PMC9966977; doi:10.3390/insects14020118)
Supplement: Supplementary file 1 [file insects-14-00118-s001.zip › insects-2065559_R2_Supplementary_Material.pdf]

Supplementary file: Are ants good organisms to teach elementary students about invasive species? (Zollota et al., Insects)

Quentin D. Read

December 19, 2022

- Summary
  - Description of methods
  - Research questions
  - Abstracted results
- Setup
- Statistical analysis
  - Fit models
  - Check model diagnostics
  - Estimate marginal means
  - Contrasts and interaction contrasts
- Results
  - Knowledge
  - Attitude

Summary

Description of methods

In this document, I fit two separate logistic regressions to answer the research questions described below. One model is for changes in knowledge about ants and invasive species, and the other is for changes in attitude about ants and about care for the environment resulting from the education program. The models also allow us to ask whether the changes were different between different categories of question, or between the two schools where the program was done. Estimated marginal means were calculated from the model, contrasts and interaction contrasts were taken, and the results are presented in graphs and tables.

Research questions

- Was there any change in knowledge about ants or about invasive species in general as a result of the education program? (Within each school separately and averaged across schools)
- Was there any change in attitude about ants or about care for the environment as a result of the education program? (Within each school separately and averaged across schools)
- Was the knowledge/attitude about ants both before and after the program different between the two schools?
- Was the effect of the program different between the two schools?

Abstracted results

There is a very strong trend in increasing knowledge of ants and invasive species as a result of the program. Knowledge about invasive species increased more than knowledge about ants. These trends were similar between the two schools.

There is also a strong trend in increasing positive attitude about ants and care for the environment as a result of the program. Positive attitudes about care for the environment increased slightly more than positive attitudes about ants. These trends were similar between the two schools.

Before the program, ME students had significantly higher knowledge about ants than LE students. This difference was smaller after the program. Both before and after the program, ME students had significantly more positive attitude about ants and caring for the environment.

Setup

```
library(tidyverse)
library(emmeans)
library(gt)
library(glue)
library(performance)
```

Load the data.

```
survey_data <- read_csv('data/ascunce/ant_survey_clean.csv') %>%
  mutate(survey = factor(survey, levels = c('pre', 'post'), labels = c('before', 'after')))
```

For questions in the knowledge categories that have a right or wrong answer, combine the wrong and “do not know” answers together.

```
knowledge <- survey_data %>%
  filter(category %in% c('ant knowledge', 'general impact knowledge', 'invasive species knowledg
e')) %>%
  mutate(right = if_else(correct_answer == 'yes', yes, no),
         wrong = if_else(correct_answer == 'yes', no + do_not_know, yes + do_not_know))
```

Subset the attitude questions as well. All the attitude questions are “positive” so we can model change in the frequency of “yes” answers. Sum up the no and “do not know” answers.

```
attitude <- survey_data %>%
  filter(category %in% c('general care for environment', 'general feelings about ants')) %>%
  mutate(not_yes = no + do_not_know)
```

Statistical analysis

Fit models

Fit a generalized linear model (logistic regression or binomial GLM) using survey (before or after), category of questions, and school as effects. Also include all two-way and three-way interactions. Do a separate model for the knowledge questions and the attitude questions.

```
glm_knowledge <- glm(cbind(right, wrong) ~ survey * category * school, data = knowledge, family
= binomial)
glm_attitude <- glm(cbind(yes, not_yes) ~ survey * category * school, data = attitude, family =
binomial)
```

Check model diagnostics

To ensure that the residuals meet the assumptions of normal distribution and homogeneous variance, make diagnostic plots. Inspecting the plots shows that the model assumptions are met (no overall trend in square root of standardized residuals versus fitted values and no major deviations from straight line for normal Q-Q plot).

Diagnostic plots: knowledge model

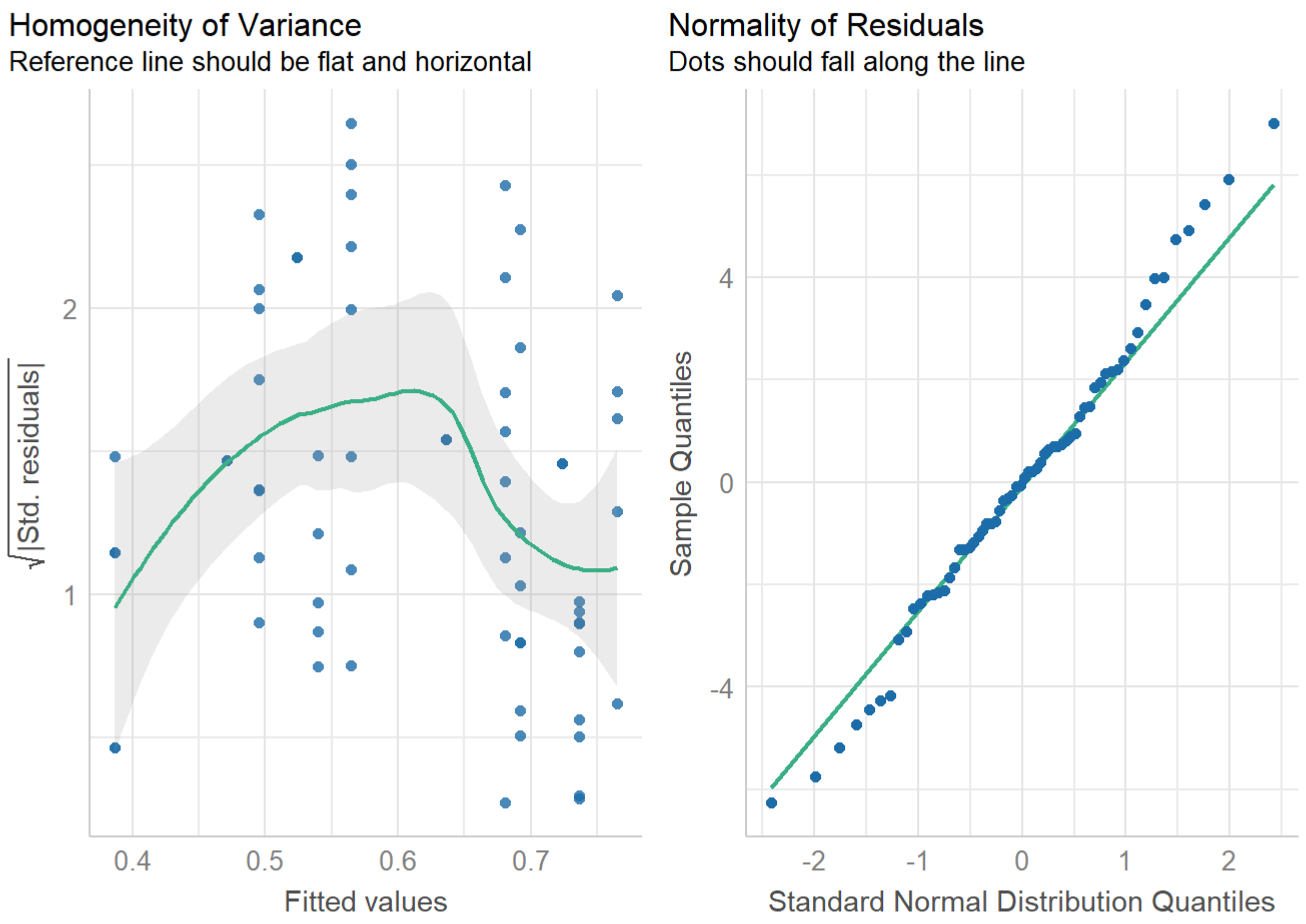

Diagnostic plots: attitude model

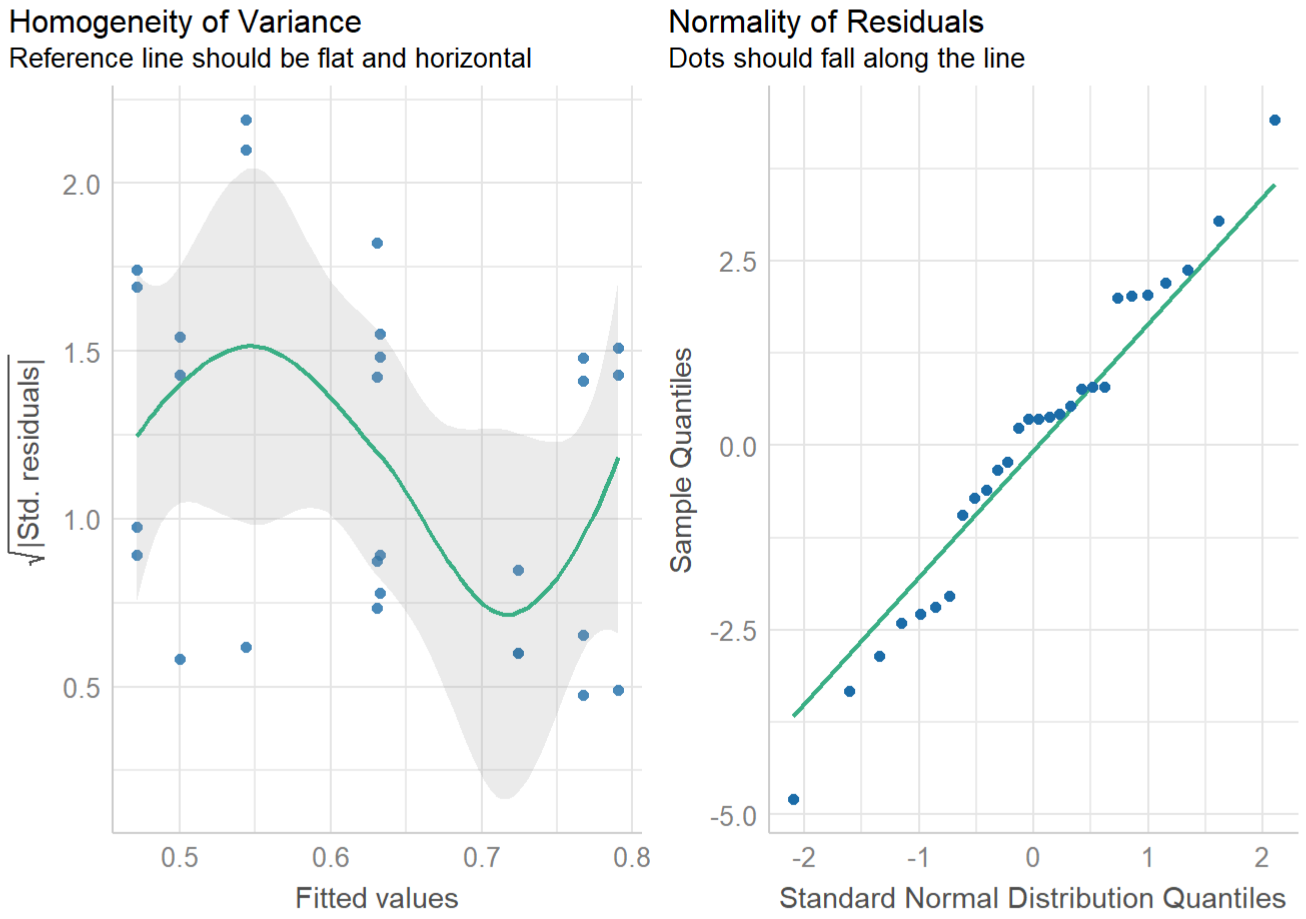

Estimate marginal means

For each GLM, estimate the marginal means of predicted percent correct (for knowledge questions) or percent positive attitude (for attitude questions) for the following:

- Before and after, averaged across both schools and all question categories
- Before and after for each category, averaged across both schools
- Before and after for each school, averaged across all question categories
- Before and after for each category separately within each school

For each mean, a 95% confidence interval is estimated based on the z-statistic.

```
comparisons <- list(survey = ~ survey,
                    survey_by_category = ~ survey + category,
                    survey_by_school = ~ survey + school,
                    three_way = ~ survey + category + school)

emm_knowledge <- emmeans(glm_knowledge, comparisons, type = 'response')
emm_attitude <- emmeans(glm_attitude, comparisons, type = 'response')
```

Contrasts and interaction contrasts

Do a statistical test (z-test) testing whether the odds ratio is significantly different from 1 for the following comparisons. The before-after contrasts are odds ratios because we are looking at the difference between two different probabilities: probability of a correct or positive answer before the program and after the program. The interaction contrasts are a ratio of two odds ratios. This is done for both the knowledge questions and the attitude questions. When comparing more than two means, a Sidak adjustment is made for multiple comparisons. In all cases, 1 is the null value representing no change (equal ratio).

- Is the odds of correct answer/positive attitude greater after the test than before, averaged across both schools and question categories?
- Is the odds of correct answer/positive attitude different between schools, averaged across categories, in the before and after surveys separately?
- Is the trend of increase in correct answer/positive attitude different between question category, averaged across schools?
- Is the trend of increase in correct answer/positive attitude different between schools, averaged across question categories?
- Is the trend of increase in correct answer/positive attitude different between schools within each question category separately?

```
contr_knowledge <- list(
  survey = emmeans(glm_knowledge, ~ survey, type = 'response') |> contrast('revpairwise'),
  school_within_survey = emmeans(glm_knowledge, ~ school | survey, type = 'response') |> contrast('revpairwise'),
  survey_by_category = emmeans(glm_knowledge, ~ survey + category, type = 'response') |> contrast(interaction = 'revpairwise', adjust = 'sidak'),
  survey_within_category_ME = emmeans(glm_knowledge, ~ survey | category, type = 'response', at = list(school = 'ME')) |> contrast(interaction = 'revpairwise') |> rbind(adjust = 'sidak'),
  survey_within_category_LE = emmeans(glm_knowledge, ~ survey | category, type = 'response', at = list(school = 'LE')) |> contrast(interaction = 'revpairwise') |> rbind(adjust = 'sidak'),
  survey_by_school = emmeans(glm_knowledge, ~ survey + school, type = 'response') |> contrast(interaction = 'revpairwise'),
  three_way = emmeans(glm_knowledge, ~ survey + school | category, type = 'response') |> contrast(interaction = 'revpairwise') |> rbind(adjust = 'sidak')
)

contr_attitude <- list(
  survey = emmeans(glm_attitude, ~ survey, type = 'response') |> contrast('revpairwise'),
  school_within_survey = emmeans(glm_attitude, ~ school | survey, type = 'response') |> contrast('revpairwise'),
  survey_by_category = emmeans(glm_attitude, ~ survey + category, type = 'response') |> contrast(interaction = 'revpairwise', adjust = 'sidak'),
  survey_within_category_ME = emmeans(glm_attitude, ~ survey | category, type = 'response', at = list(school = 'ME')) |> contrast(interaction = 'revpairwise') |> rbind(adjust = 'sidak'),
  survey_within_category_LE = emmeans(glm_attitude, ~ survey | category, type = 'response', at = list(school = 'LE')) |> contrast(interaction = 'revpairwise') |> rbind(adjust = 'sidak'),
  survey_by_school = emmeans(glm_attitude, ~ survey + school, type = 'response') |> contrast(interaction = 'revpairwise'),
  three_way = emmeans(glm_attitude, ~ survey + school | category, type = 'response') |> contrast(interaction = 'revpairwise') |> rbind(adjust = 'sidak')
)
```

Results

Knowledge

This figure shows the knowledge GLM model's estimates of percent correct scores for each category and each school separately, as well as averaged across categories overall and averaged across schools overall. Points show the estimated marginal means, colored by survey (before or after), and error bars show the 95% confidence intervals of the means.

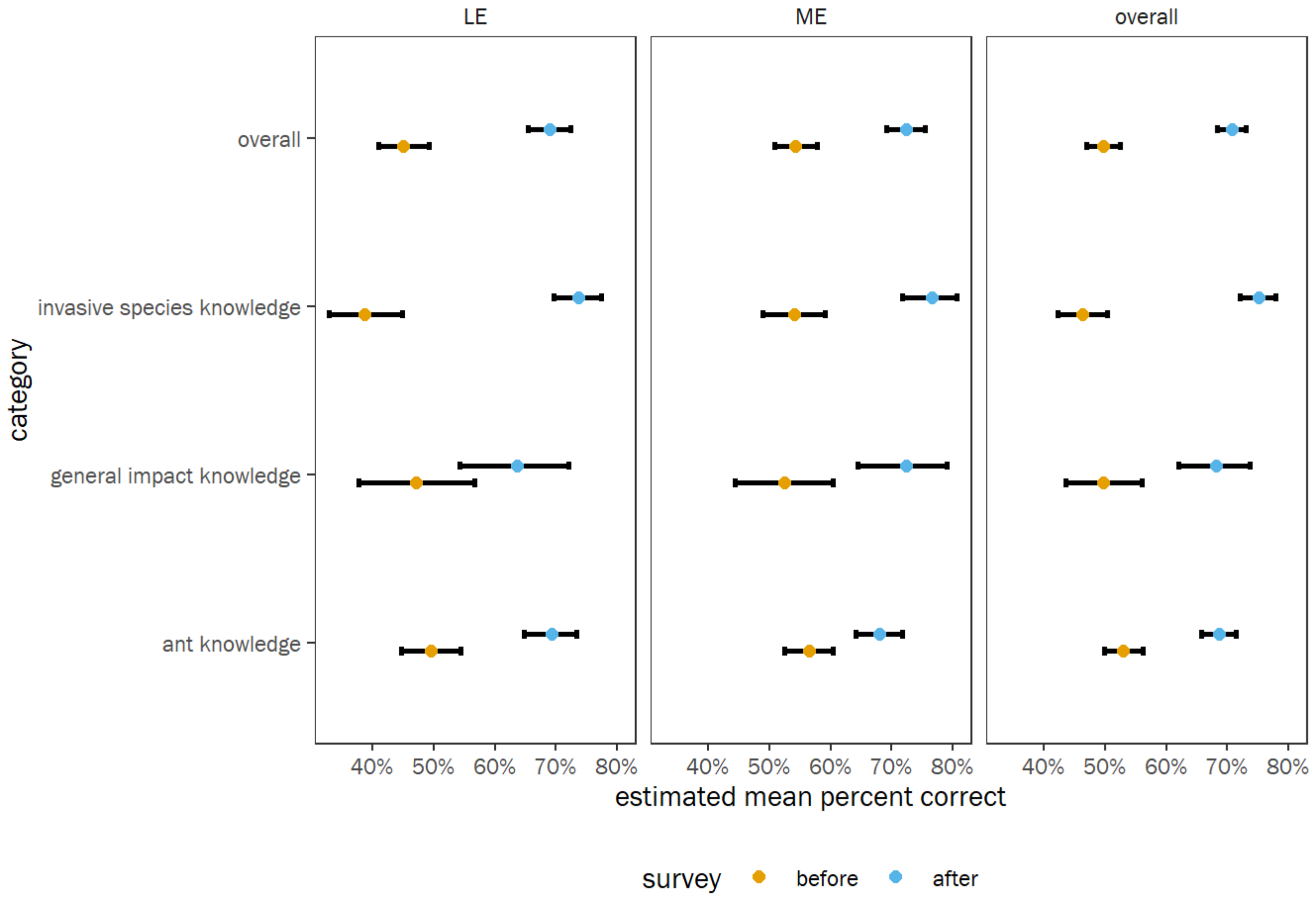

This table is the same information as contained in the figure above (estimated marginal means and 95% confidence intervals).

survey	category	estimated proportion correct	95% confidence interval
**overall**			
before	overall	0.497	(0.47, 0.524)
after	overall	0.707	(0.683, 0.73)
before	ant knowledge	0.530	(0.498, 0.562)
after	ant knowledge	0.686	(0.657, 0.714)

survey	category	estimated proportion correct	95% confidence interval
before	general impact knowledge	0.498	(0.435, 0.56)
after	general impact knowledge	0.681	(0.621, 0.737)
before	invasive species knowledge	0.462	(0.422, 0.503)
after	invasive species knowledge	0.751	(0.72, 0.78)
**LE**			
before	overall	0.450	(0.41, 0.492)
after	overall	0.690	(0.655, 0.723)
before	ant knowledge	0.495	(0.447, 0.543)
after	ant knowledge	0.692	(0.648, 0.733)
before	general impact knowledge	0.471	(0.377, 0.567)
after	general impact knowledge	0.636	(0.543, 0.721)
before	invasive species knowledge	0.387	(0.329, 0.448)
after	invasive species knowledge	0.737	(0.696, 0.773)
**ME**			
before	overall	0.543	(0.508, 0.577)
after	overall	0.724	(0.691, 0.755)
before	ant knowledge	0.565	(0.524, 0.605)
after	ant knowledge	0.680	(0.641, 0.718)
before	general impact knowledge	0.524	(0.443, 0.604)
after	general impact knowledge	0.723	(0.644, 0.791)
before	invasive species knowledge	0.540	(0.488, 0.591)
after	invasive species knowledge	0.765	(0.718, 0.807)

This table presents the statistical test results for the odds ratio contrasts and z-tests. Again, an odds ratio of 1 indicates no change. We see significantly greater proportion correct scores after the program versus before, averaged across all categories and schools (OR = 2.45 times greater odds of correct scores, $p < 0.0001$). In the pre-test, ME had significantly higher knowledge than LE (OR = 1.45, $p = 0.0008$), but the difference was less and not significantly different in the post-test (OR = 1.18, $p = 0.15$). When comparing the trend between question categories, we see that invasive species knowledge increased more than ant knowledge (OR = 1.81, $p = 0.0002$). All trends were significantly positive when looking at each category and school individually. The trend was similar between the two schools ($p > 0.05$ indicating OR is not significantly different from 1), and the trend within each question category was similar between the two schools ($p > 0.05$ in all cases indicating OR is not significantly different from 1).

comparison	odds ratio	standard error	z ratio	p-value
after/before ratio, averaged across all categories and schools	2.450	0.196	11.209	3.7×10^{-29}
ME/LE ratio, pre-test	1.449	0.161	3.348	0.000813
ME/LE ratio, post-test	1.181	0.136	1.442	0.149
trend comparison general impact knowledge vs. ant knowledge, averaged across schools	1.113	0.234	0.511	0.94
trend comparison invasive species knowledge vs. ant knowledge, averaged across schools	1.808	0.270	3.971	0.000215
trend comparison invasive species knowledge vs. general impact knowledge, averaged across schools	1.624	0.358	2.199	0.0814
after/before ratio, ant knowledge, ME only	1.640	0.202	4.009	0.000183
after/before ratio, general impact knowledge, ME only	2.374	0.596	3.443	0.00173
after/before ratio, invasive species knowledge, ME only	2.774	0.456	6.207	1.63×10^{-9}
after/before ratio, ant knowledge, LE only	2.294	0.325	5.861	1.38×10^{-8}
after/before ratio, general impact knowledge, LE only	1.964	0.548	2.419	0.0459
after/before ratio, invasive species knowledge, LE only	4.436	0.724	9.120	0
trend comparison ME vs. LE, averaged across all categories	0.815	0.130	−1.283	0.2
trend comparison ME vs. LE, ant knowledge category	0.715	0.134	−1.786	0.206

comparison	odds ratio	standard error	z ratio	p-value
trend comparison ME vs. LE, general impact knowledge category	1.209	0.454	0.505	0.942
trend comparison ME vs. LE, invasive species knowledge category	0.625	0.145	−2.025	0.123

Attitude

This figure shows the attitude GLM model's estimates of percent positive attitude scores for each category and each school separately, as well as averaged across categories overall and averaged across schools overall.

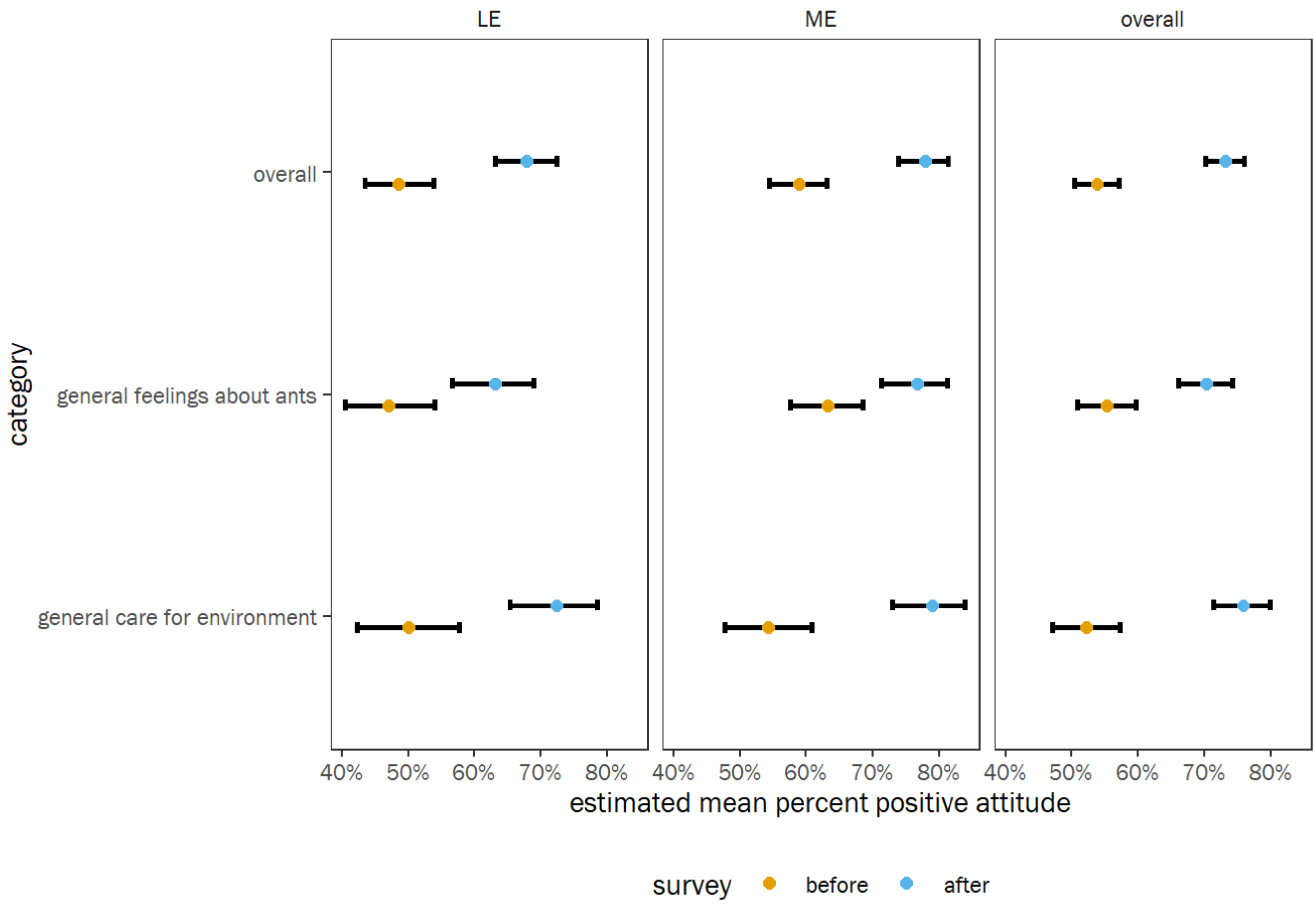

This table is the same information as contained in the figure above (estimated marginal means and 95% confidence intervals).

survey	category	estimated proportion positive	95% confidence interval
**overall**			
before	overall	0.538	(0.503, 0.571)

survey	category	estimated proportion positive	95% confidence interval
after	overall	0.732	(0.701, 0.761)
before	general care for environment	0.522	(0.47, 0.573)
after	general care for environment	0.759	(0.713, 0.799)
before	general feelings about ants	0.553	(0.508, 0.597)
after	general feelings about ants	0.703	(0.662, 0.742)
**LE**			
before	overall	0.486	(0.434, 0.537)
after	overall	0.679	(0.631, 0.724)
before	general care for environment	0.500	(0.422, 0.578)
after	general care for environment	0.724	(0.653, 0.785)
before	general feelings about ants	0.471	(0.405, 0.539)
after	general feelings about ants	0.630	(0.566, 0.69)
**ME**			
before	overall	0.589	(0.545, 0.631)
after	overall	0.779	(0.739, 0.814)
before	general care for environment	0.543	(0.477, 0.608)
after	general care for environment	0.790	(0.73, 0.84)
before	general feelings about ants	0.632	(0.575, 0.686)
after	general feelings about ants	0.767	(0.714, 0.813)

This table presents the statistical test results for the odds ratio contrasts and z-tests. Again, an odds ratio of 1 indicates no change. We see significantly greater proportion positive attitude scores after the program versus before, averaged across all categories and schools (OR = 2.35 times greater odds of positive attitude scores, $p < 0.0001$). ME had significantly more positive attitude than LE in the pre-test (OR = 1.52, $p = 0.003$) as well as the post-test (OR = 1.67, $p = 0.001$). When comparing the trend between question categories, we see weak evidence that positive feelings about care for the environment increased more than positive feelings about ants (OR = 1/0.663 = 1.51 times greater increase in positive feelings about environment relative to ants, $p = 0.049$). However all trends were significantly positive when looking at each category and school individually. The trend was similar

between the two schools (p > 0.05 indicating OR is not significantly different from 1), and the trend within each question category was similar between the two schools (p > 0.05 in both cases indicating OR is not significantly different from 1).

comparison	odds ratio	standard error	z ratio	p-value
after/before ratio, averaged across all categories and schools	2.350	0.245	8.187	2.67×10^{-16}
ME/LE ratio, pre-test	1.515	0.211	2.975	0.00293
ME/LE ratio, post-test	1.665	0.258	3.289	0.00101
trend comparison feelings about ants vs. feelings about care for environment, averaged across schools	0.663	0.138	−1.967	0.0492
after/before ratio, feelings about ants, ME only	3.170	0.688	5.314	2.15×10^{-7}
after/before ratio, feelings about care for environment, ME only	1.915	0.357	3.480	0.001
after/before ratio, feelings about ants, LE only	2.625	0.612	4.137	7.03×10^{-5}
after/before ratio, feelings about care for environment, LE only	1.913	0.372	3.337	0.00169
trend comparison ME vs. LE, averaged across all categories	1.100	0.229	0.455	0.649
trend comparison ME vs. LE, feelings about ants category	1.208	0.385	0.592	0.801
trend comparison ME vs. LE, feelings about care for environment category	1.001	0.270	0.004	1

school	survey	category	question	correct_answer	no	yes	do_not_know
LE	pre	ant knowledge	Ants bodies have three parts	yes	11	32	9
LE	post	ant knowledge	Ants bodies have three parts	yes	14	41	3
ME	pre	ant knowledge	Ants bodies have three parts	yes	2	57	14
ME	post	ant knowledge	Ants bodies have three parts	yes	19	51	1
LE	pre	ant knowledge	Ants use chemical signals to communicate with each other	yes	14	23	15
LE	post	ant knowledge	Ants use chemical signals to communicate with each other	yes	8	45	5
ME	pre	ant knowledge	Ants use chemical signals to communicate with each other	yes	6	36	30
ME	post	ant knowledge	Ants use chemical signals to communicate with each other	yes	10	37	23
LE	pre	ant knowledge	Fire ant stings are dangerous	yes	24	15	12
LE	post	ant knowledge	Fire ant stings are dangerous	yes	13	39	6
ME	pre	ant knowledge	Fire ant stings are dangerous	yes	21	32	19
ME	post	ant knowledge	Fire ant stings are dangerous	yes	21	38	10
LE	pre	ant knowledge	Fire ants are a problem	yes	17	19	15
LE	post	ant knowledge	Fire ants are a problem	yes	8	41	7
ME	pre	ant knowledge	Fire ants are a problem	yes	15	39	19
ME	post	ant knowledge	Fire ants are a problem	yes	10	46	12
LE	pre	ant knowledge	Fire ants are an invasive species	yes	11	21	19
LE	post	ant knowledge	Fire ants are an invasive species	yes	14	36	7
ME	pre	ant knowledge	Fire ants are an invasive species	yes	14	18	40
ME	post	ant knowledge	Fire ants are an invasive species	yes	10	53	8
LE	pre	ant knowledge	Fire Ants love sugar	yes	24	11	16
LE	post	ant knowledge	Fire Ants love sugar	yes	24	22	10
ME	pre	ant knowledge	Fire Ants love sugar	yes	28	16	28
ME	post	ant knowledge	Fire Ants love sugar	yes	22	32	17
LE	pre	ant knowledge	I think it is ok to touch a fire ant pile	no	38	6	6
LE	post	ant knowledge	I think it is ok to touch a fire ant pile	no	41	12	3
ME	pre	ant knowledge	I think it is ok to touch a fire ant pile	no	60	11	1
ME	post	ant knowledge	I think it is ok to touch a fire ant pile	no	54	11	4
LE	pre	ant knowledge	There is only one type of ant	no	44	5	3
LE	post	ant knowledge	There is only one type of ant	no	50	5	1
ME	pre	ant knowledge	There is only one type of ant	no	69	2	2
ME	post	ant knowledge	There is only one type of ant	no	70	1	0
LE	pre	general care for environment	I help the environment on a daily basis		13	25	14
LE	post	general care for environment	I help the environment on a daily basis		9	40	9
ME	pre	general care for environment	I help the environment on a daily basis		22	23	28
ME	post	general care for environment	I help the environment on a daily basis		7	49	14
LE	pre	general care for environment	I put effort into taking care of native species		12	20	20
LE	post	general care for environment	I put effort into taking care of native species		6	43	9
ME	pre	general care for environment	I put effort into taking care of native species		18	41	14
ME	post	general care for environment	I put effort into taking care of native species		9	56	5
LE	pre	general care for environment	I try to protect the environment and ecosystems around me		8	33	11
LE	post	general care for environment	I try to protect the environment and ecosystems around me		6	43	9
ME	pre	general care for environment	I try to protect the environment and ecosystems around me		6	55	12
ME	post	general care for environment	I try to protect the environment and ecosystems around me		4	61	5
LE	pre	general feelings about ants	Are ants fun to learn about?		15	22	16
LE	post	general feelings about ants	Are ants fun to learn about?		12	39	7
ME	pre	general feelings about ants	Are ants fun to learn about?		7	44	22
ME	post	general feelings about ants	Are ants fun to learn about?		4	59	6
LE	pre	general feelings about ants	Are ants important?		10	34	8
LE	post	general feelings about ants	Are ants important?		6	43	9
ME	pre	general feelings about ants	Are ants important?		6	49	18
ME	post	general feelings about ants	Are ants important?		7	55	8
LE	pre	general feelings about ants	Do you like ANTS?		29	16	8
LE	post	general feelings about ants	Do you like ANTS?		27	26	5
ME	pre	general feelings about ants	Do you like ANTS?		26	37	9
ME	post	general feelings about ants	Do you like ANTS?		14	47	9
LE	pre	general feelings about ants	Do you want to learn more about Ants?		14	27	11
LE	post	general feelings about ants	Do you want to learn more about Ants?		13	37	6
ME	pre	general feelings about ants	Do you want to learn more about Ants?		6	54	13
ME	post	general feelings about ants	Do you want to learn more about Ants?		7	53	10
LE	pre	general impact knowledge	Invasive species cost our government a lot of money in damages	yes	8	19	25
LE	post	general impact knowledge	Invasive species cost our government a lot of money in damages	yes	10	29	16
ME	pre	general impact knowledge	Invasive species cost our government a lot of money in damages	yes	16	24	33
ME	post	general impact knowledge	Invasive species cost our government a lot of money in damages	yes	8	45	17
LE	pre	general impact knowledge	The balance of the species in our ecosystem is important	yes	9	30	13
LE	post	general impact knowledge	The balance of the species in our ecosystem is important	yes	6	41	8
ME	pre	general impact knowledge	The balance of the species in our ecosystem is important	yes	10	52	10

school	survey	category	question	correct_answer	no	yes	do_not_know
ME	post	general impact knowledge	The balance of the species in our ecosystem is important	yes	6	57	8
LE	post	invasive species knowledge	Affecting one area of our ecosystem can have a rippling effects on other parts	yes	4	39	11
LE	pre	invasive species knowledge	Ants interact with the ecosystem as much as any other animal	yes	7	20	23
LE	post	invasive species knowledge	Ants interact with the ecosystem as much as any other animal	yes	8	39	10
ME	pre	invasive species knowledge	Ants interact with the ecosystem as much as any other animal	yes	8	41	23
ME	post	invasive species knowledge	Ants interact with the ecosystem as much as any other animal	yes	13	41	17
LE	post	invasive species knowledge	Can you explain to your friend what native and invasive species are?	yes	10	41	5
LE	post	invasive species knowledge	I know how some invasive species are introduced	yes	10	38	7
LE	post	invasive species knowledge	I know how some invasive species can harm the enviroment	yes	8	43	4
LE	pre	invasive species knowledge	I know what a native species is	yes	26	16	10
LE	post	invasive species knowledge	I know what a native species is	yes	8	44	4
ME	pre	invasive species knowledge	I know what a native species is	yes	21	43	9
ME	post	invasive species knowledge	I know what a native species is	yes	6	62	1
LE	pre	invasive species knowledge	I know what an Invasive species is	yes	27	16	9
LE	post	invasive species knowledge	I know what an Invasive species is	yes	8	44	5
ME	pre	invasive species knowledge	I know what an Invasive species is	yes	26	31	16
ME	post	invasive species knowledge	I know what an Invasive species is	yes	7	61	1
LE	pre	invasive species knowledge	Invasive ants compete with native ants for food and resources	yes	9	20	21
LE	post	invasive species knowledge	Invasive ants compete with native ants for food and resources	yes	5	41	11
ME	pre	invasive species knowledge	Invasive ants compete with native ants for food and resources	yes	9	36	27
ME	post	invasive species knowledge	Invasive ants compete with native ants for food and resources	yes	8	49	14
LE	pre	invasive species knowledge	Protecting native ants is important for the enviroment	yes	4	27	21
LE	post	invasive species knowledge	Protecting native ants is important for the enviroment	yes	8	43	7
ME	pre	invasive species knowledge	Protecting native ants is important for the enviroment	yes	9	45	19
ME	post	invasive species knowledge	Protecting native ants is important for the enviroment	yes	5	54	10
